# Intracorneal Stromal Ring Can Affect the Biomechanics of Ectatic Cornea

**DOI:** 10.1155/2020/4274037

**Published:** 2020-10-07

**Authors:** Shahram Bamdad, Mohammad Reza Sedaghat, Masoud Yasemi, Mehrnoosh Maalhagh

**Affiliations:** ^1^Poostchi Ophthalmology Research Center, Department of Ophthalmology, School of Medicine, Shiraz University of Medical Sciences, Shiraz, Iran; ^2^Department of Ophthalmology, School of Medicine, Mashhad University of Medical Sciences, Mashhad, Iran

## Abstract

**Purpose:**

The biomechanical properties of ecstatic cornea undergo changes. This study evaluates the biomechanical changes of ecstatic cornea after implantation of two types of intracorneal stromal ring (ICR).

**Methods:**

For doing this prospective cross-sectional study, 32 patients with keratoconus (KCN) were randomly divided into two 16-member groups (group I: MyoRing, group II: KeraRing). The main inclusion criteria were transparent cornea with no scar in the central part, corneal thickness >450 *µ* in the incision region, keratometry within 48–52 diopters, and progressive course of corneal thinning. Biomechanics of the cornea was evaluated by “ORA” and “Corvis” devices. All of the data were recorded and analyzed before implantation of the rings and 6 months thereafter.

**Results:**

The mean ages of patients of groups I and II were 26 ± 6.55 and 33.86 ± 8.5, respectively. The postoperative change of sphere refraction was significant in both groups. However, reduction in the astigmatism was significant only in group I. In addition, the change of flat meridian keratometry (Kf) was significant before and after ring implantation in group I, unlike group II. The changes in CH and CRF parameters (ORA) were not significant in either group before and after the operation. Besides, only HRC parameter (Corvis) decreased significantly in both groups before and after the operation.

**Conclusion:**

Both MyoRing and KeraRings have positive effects on the biomechanics of cornea at least during the first year after implantation. Comparison of these two types of ICR did not show significant differences in Corvis and ORA parameters.

## 1. Introduction

Keratoconus (KCN) is a corneal disorder causing a progressive noninflammatory status and, eventually, gradual thinning of the cornea along with progression of myopia and astigmatism [1, 2]. In such cases, implantation of an intracorneal stromal ring (ICR) is a relatively safe and reversible method for improving vision, through which no tissue is excised from the cornea, and the center of it is not manipulated [3, 4]. KeraRings and MyoRings are two common types of ICR. KeraRings are made of poly (methyl methacrylate) (PMMA) and implanted in the corneal stroma, causing diminished astigmatism and myopia by changing the central curvature and corneal shape [2]. MyoRings are also made of PMMA and are implanted in a pocket in the cornea as a complete 360° ring [3]. In patients with KCN, the biomechanical properties of the cornea undergo changes because of progressive corneal thinning. The biomechanical properties of the cornea are measured by examining the corneal response to pressure and are dependent on the internal consistency of the cornea [4, 5]. In recent years, various instruments for measuring the biomechanical properties of the cornea have been designed, including the ocular response analyzer (ORA) and corneal visualization Scheimpflug technology (Corvis-ST, CST) [6].

ORA examines the corneal dynamics by merging two methods: applanation and air puff. With ORA, the necessary pressure for flattening the cornea in inward and outward directions is measured, and the difference between these two pressures is reported as the corneal hysteresis (CH). The other variable is the corneal resistance factor (CRF), which is calculated by linear equation and reports the elastic properties of the cornea [6, 7]. The CST method is a quantitative and qualitative method for measuring the biomechanical properties of the cornea as well as a noncontact tonometry which tests the dynamic response of the cornea to air impulse. It also measures the extent of changes in the corneal height inwards after applying the air impulse [7]. CST measures difference variables, including corneal thickness, range of corneal changes, applanation length, corneal deformity changes, and anterior corneal curvature [7, 8]. The aims of the current study were to investigate the biomechanical changes of corneas among KCN patients after ICRS implantation and to compare the results of KeraRing and MyoRing implantation.

## 2. Materials and Methods

### 2.1. Study Setting and Ethical Considerations

This prospective cross-sectional study, conducted in 2017–2019, investigated 32 KCN patients within the age ranges of 15–35 and 18–46 in two groups of patients undergoing KeraRing and MyoRing treatment, respectively. Sixteen patients were treated by KeraRing implantation, while 16 others received MyoRing implants. Patient information was collected in the Ophthalmology Clinic of Mashhad University of Medical Sciences (MUMS). This research project was approved by the Ethics Committee of MUMS, and all patients granted informed consent for participation prior to inclusion, based on the Helsinki declaration.

### 2.2. Eligibility Criteria

Among the patients with definite KCN, inclusion criteria for ICRS implantation were intolerance to spectacles or rigid gas permeable contact lenses (RGP), a transparent cornea with no scarring in the central part, corneal thickness greater than 450 microns in the incision region, keratometry between 48 and 52 diopters (D), and no therapeutic option other than corneal transplantation to improve their functional vision. The contraindications of ICRS implantation in KCN patients were keratometry over 70 D, central corneal opacity (in the vision axis), existence of an irregular scar in the cornea, patients with atopic and chronic itching, consumption of immunomodulatory drugs, ocular infections, rheumatologic diseases, and corneal dystrophia [9]. Patients with spherical equivalents greater than 5 were selected for MyoRing implantation, while patients with astigmatism dominancy with a lower spherical equivalent were selected for KeraRing implantation.

### 2.3. Patient Enrollment

KCN was diagnosed based on the presence of the following signs and symptoms in the examination: irregular cornea (based on based on retinoscopy examination) or red reflex distortion (scissor reflex) in retinoscopic examination and the presence of astigmatism [10]. For final confirmation, Pentacam images were taken from all patients (Scheimpflug). All patients were forbidden to wear hard or soft contact lenses for one month. At the beginning of the study, all patients underwent complete ophthalmology examinations including cycloplegic refraction (CR), uncorrected distance visual acuity (UCDVA), best corrected visual acuity (BCVA), biomechanical evaluation of the cornea by CST and ORA, and evaluation of the topography and thickness of the cornea by Scheimpflug pachymetry (Pentacam). To achieve exact results, at least three images and scans for used modalities were obtained for each patient. All examinations and paraclinical measures were done between 8 and 11 a.m. by one ophthalmologist and the same operator at the ophthalmology clinic of MUMS.

The parameters that were evaluated by Pentacam, CST, and ORA are reported in Table 1. These parameters were measured and recorded in the patients' files both before ring implantation surgery and 6 months thereafter. All ring implantation operations were done by a cornea subspecialist.

### 2.4. Surgical Method

Procedures were performed under general local anesthesia. Povidone iodine (Betadine) 5% and 10% was used to sterilize the eye, the eyelids, and the surrounding skin, respectively. After inserting the lid speculum, pocket formation was done by femtosecond laser (Ziemer Company, FEMTO Z6 model, Switzerland).

#### 2.4.1. MyoRing Implantation

After the pocket was made, a space was carefully formed using a spatula through the temporal incision between the closed flap and the bed. Then the MyoRing, selected based on the standard nomogram of Daxer et al. [11], was placed into the corneal tunnel through the temporal incision. Finally, a soft contact lens was applied.

#### 2.4.2. KeraRing Implantation

The tunnel was created using a femtosecond laser (Ziemer Company, Femto Z6 model, Switzerland), and the KeraRing was inserted according to the Mediphacos [12] nomogram. Finally, a bandage contact lens was applied.

### 2.5. Follow-Up

An examination was performed one day after surgery using a slit lamp, and topical ciprofloxacin and betamethasone (one drop every six hours) were prescribed for one month. Subsequent follow-up examinations were done at two weeks, one month, 3 months, and 6 months after surgery. Among the follow-ups, danger signs including eye redness, foreign body sensation, and pussy discharge were explained to the patients, and they were told to refer to the clinic in case of the incidence of any of these symptoms. If any surgical complication occurred, the patient would be excluded from the study. In the 6-month follow-up examination, visual acuity was tested, a slit lamp examination was performed, and the cornea topography was examined by Pentacam. Moreover, a corneal biomechanical examination was done by CST and ORA postoperation. The results of these examinations and imaging were then compared with preoperation results.

### 2.6. Statistical Analysis

Data was statistically analyzed using SPSS 18. To investigate the normal distribution of data, the Kolmogorov–Smirnov test was used. Eventually, data with normal distribution and that with abnormal distribution were compared using the Student sample *t*-test and Mann–Whitney test, respectively. The significance level of tests was considered as *p* value <0.05.

## 3. Results

Overall, this study examined 32 patients: 16 who received MyoRing implants (group 1), and 16 who received KeraRing implants (group 2). The mean ages of the two groups were 26 ± 6.55 and 33.86 ± 8.5 years, respectively. The pre-/post-ICR implantation results in both groups were inspected in terms of refraction, topography, and biomechanical changes of the cornea. When comparing the two groups before ring implantation, no significant difference was found in any of the parameters.

### 3.1. Refractive Outcomes

In the patients treated with a MyoRing implant (group 1), the changes in sphere refraction were statistically significant before and after surgery (6 months) and showed a reduction in the extent of sphericity (*p* value = 0.001). In patients treated with a KeraRing implant (group 2), the sphere refraction changes were statistically significant before and after surgery (6 months) (*p* value = 0.039). The changes in cylinder refraction 6 months after ring implantation were significantly reduced in group 1 patients, but not in group 2. Nevertheless, comparing groups 1 and 2, we found that these changes were not significant (*p* value = 0.59).

### 3.2. Keratometry and Corneal Thickness (Pentacam)

In group 1, the changes in CCT before and 6 months after the operation were not significant (*pvalue* = 0.75); however, the changes in corneal thickness were significant in the central 4 mm (*p* value = 0.025). Moreover, the changes in the keratometry of the flat meridian (Kf) before and after ring implantation were significant in group 1 (*p* value = 0.008). In group 2, the changes in CCT before and after surgery (*pvalue* = 0.75) were not significant. The variations in the Ks and Kf values were not significant either before or 6 months after surgery. When comparing the results of corneal thickness, no significant difference was observed between the two groups after surgery (*p* value >0.05).

### 3.3. Biomechanical Changes (ORA/CORVIS)

The corneal biomechanical changes were examined by ORA and CST parameters. The variations in the CRF and CH parameters were not significant in group 1 before or after surgery (*pvalue* = 0.067). Similar results were obtained when comparing CRF and CH before and after surgery in group 2 (*pvalue* = 0.36). A comparison of the two groups 6 months after ring implantation showed that, among the CST parameters, only the highest radius of curvature was significantly diminished (*p* value = 0.001) in both groups before and after surgery. The parameters of groups 1 (MyoRing) and 2 (KeraRing) before and after ICRS implantation are shown in Tables 2 and 3, respectively.

### 3.4. Intraocular Pressure (IOP) Changes

The changes in postoperative intraocular pressure were not significant in group 1, unlike group 2; IOP was significantly less after than before operation in the KeraRing group based on Corvis/ORA measurements.  Table 4 shows the results of the evaluated parameters before and after ICRS implantation in each group and in comparison with each other.

## 4. Discussion

The biomechanical properties of the cornea include elasticity, viscosity, and viscoelasticity, which indeed represent the corneal response to the compressive force exerted on the cornea [8]. These properties are influenced by different factors, including age, corneal hydration, corneal thickness, arrangement of collagen fibrils, and integrity of the epithelial barrier as well as some underlying diseases such as diabetes mellitus [13, 14]. CST and ORA devices examine different parameters to test the biomechanical properties of the cornea. So far, biomechanical changes of the cornea have been measured with these two devices in KCN and diabetic patients as well as those undergoing refractive surgery, and they have been compared with the corneal biomechanics in normal individuals. Based on most of these studies, the corneal response parameters, including velocity, HRC (highest concavity radius), and MDA (maximum deformation amplitude), have not been significantly different between KCN patients and normal individuals, and most of these parameters have been associated with corneal thickness [1, 15]. In the present study, CRF and CH had no significant change after ICRS implantation in either group in comparison to those before operation. Similarly, in the study by Dauwe et al., no significant change was observed in CH and CRF values one year after implanting the Ferrara ring [16].

A 2011 study indicated that, following KeraRing implantation, the mean CH decreased in the first month after operation; however, in follow-up examinations one year after surgery, CH was increased and reached the same preoperative level. Two years after operation, the CH value even exceeded the preoperation value. The mean CRF had diminished significantly early after operation (3 months after surgery). Then, at the one-year postoperation follow-up, the CRF value had increased, though not statistically significantly [13]. Zare et al. examined corneal biomechanical changes after Intacs implantation. Despite reductions in CH and CRF following Intacs implantation, the changes were not statistically significant, which is in line with the results of the present study [17]. Thus, considering CH and CRF changes, the results of the present study are similar overall with previous research findings, which suggests the relative biomechanical stability of the cornea, at least at the first six-month follow-up after ICRS implantation, regardless of ring type, in KCN patients. Based on the results of previous studies, MDA in Corvis is a reliable factor in diagnosing ectatic corneal disorders, a prominent difference between KCN patients and normal individuals has been reported. Tian et al. examined the biomechanical properties of the cornea using CST in KCN patients and compared them with those of normal individuals. They observed that the MDA value was above normal in KCN patients, possibly due to poor corneal mechanical strength. In that study, from among CST parameters, MDA had the maximum sensitivity, replicability, and reliability in differentiating between the normal corneas and the corneas of patients with KCN [10]. Nevertheless, in the present study, no significant changes in MDA following ICRS implantation were seen in patients with KCN compared with their preoperation values. In contrast, significant changes were observed for the HRC parameter after ring implantation in both groups; this parameter underwent the only considerable change in the corneal biomechanics. This finding can be interpreted in different ways. If DA is considered as the reliable factor for biomechanical changes of cornea, then it can be stated that ICRS caused no significant change, at least in the six months following enhancement of the biomechanical power of the cornea. Furthermore, it may prevent its deterioration (elevation of DA value). On the other hand, significant changes in the highest radius of curvature (HRC) in both groups following ring implantation suggest the effect of ring implantation in the redistribution of the collagen lamellae arrangement in the cornea and its flattening effect on the cornea. Thus, because the effect of this rearrangement of collagen fibers of the cornea on DA requires a long-term process, the significant changes in HRC may be a prelude and relatively early effect of the ring. Confirmation of this issue requires further studies with longer follow-up times. If this finding is confirmed, the HRC factor can be used for testing the progression of corneal ectasia following ring implantation, at least in the primary stages. Steinberg et al. investigated the biomechanical changes of corneas in patients with KCN after collagen cross-linking (CXL) using CST. Their results indicated that applanation times 1 and 2 after CXL increased considerably, where in normal corneas, applanation time 1 was longer than applanation time 2, and in KCN patients, both times were diminished [18]. Based on the results of the present study, applanation times 1 and 2 in each group after ring implantation showed no significant differences in comparison with preoperation values. A comparison of both groups after ring implantation showed no significant change either. In the current study, the mean IOPs measured by ORA and CST in both groups were compared after surgery; the mean pressure measured by ORA was less than that measured by CST in both groups after operation. The mean IOP in group 1 had no significant difference from the preoperation mean. Similarly, Gorgun et al. showed that the mean IOP measured by ORA after KeraRing implantation decreased and remained at the lower level [13]. Thus, considering the impact of KeraRing implantation on ocular pressure, the ocular pressure of patients should be examined more carefully after implantation. In patients with glaucoma or ocular hypertension, the use of the MyoRing is suggested, if required. The present study showed that, after KeraRing and MyoRing implantation, CCT does not generally change; however, the CCT decreased at central 4 mm after MyoRing implantation, and this change was statistically significant. This effect might be due to the special design of the ring and its depth of implantation, as also mentioned in another study [19]. The study by Zare indicated that, after Intacs implantation, the cornea thickness increased within the first month after surgery [17]. Then, at the six-month follow-up after surgery, the thickness declined to some extent, though it did not reach the initial value [17]. The discrepancy in the results of the present study and Zare's study can be justified given the type of rings utilized (MyoRing and KeraRing vs. Intacs) and their depths of implantation (75% for Intacs and 80% for KeraRings and MyoRings). The present study also showed a significant postoperative reduction in astigmatism in group 1, unlike group 2. Thus, it seems that, for KCN patients with astigmatism greater than 1, the MyoRing is preferable over the KeraRing.

Overall, based on the results of the present study, both MyoRings and KeraRings have a positive impact on the biomechanics of the cornea at least during the first year after implantation. The comparison of these two types of ICRS showed no significant differences between Corvis and ORA in any of the examined parameters. Among the studied indicators, the only significant change was observed in Corvis-HRC. Thus, this parameter seems to be practicable for investigating the early and initial effects of ICRS on the biomechanical status of the cornea. Considering the IOP measurement, given the lack of any significant difference in the results of ORA and Corvis in the MyoRing group compared with preoperation values (unlike the KeraRing group), both instruments are suitable for assessing IOP in these patients. Note that the effects of the MyoRing in reducing astigmatism are greater than those of the KeraRing in this study.

## 5. Limitations of the Study

In this study, the IOP was not measured by Goldman tonometer. It is suggested that another study measures IOP by all three devices: Goldman, ORA, and CST after ring implantation and compares them. It is also suggested that, in another study, in addition to those suffering from KCN and undergoing ICRS implantation, healthy individuals are also investigated as the control group to compare all parameters of Corvis and ORA.

## Figures and Tables

**Table 1:** The evaluated corneal topographic and biomechanical parameters in the study.

Pentacam	ORA	CST
CCT	CH	App time 1
KF	CRF	App time 2
KS	IOP	MDA
HRC		App velocity

CCT: central corneal thickness, Kf: keratometry of flat meridian, Ks: keratometry of steep meridian, CH: corneal hysteresis, IOP: intraocular pressure, CRF: corneal resistance factor, App time 1: Applanation time 1, App time 2: Applanation time 2, MDA: maximum deformation amplitude, HRC: highest radius of curvature.

**Table 2:** The results of parameters of the group 1 (MyoRing) before and after the ICRS implantation.

MyoRing		Pre-op	Post-op	*p* value
Number		16	16	

Age		26 ± 6.55	26 ± 6.55	0.35

FCR	Sphere	−7.25 ± 4.8	−0.28 ± 5.26	0.001
	Cylinder	−3.1 ± 5.4	−0.12 ± 2.75	0.023
	Axis	102.72 ± 57.65	92.59 ± 62.10	0.79

Corvis-ST	App time 1	6.79 ± 0.30	6.98 ± 0.49	0.26
	App time 2	21.50 ± 0.46	21.52 ± 0.72	0.71
	App length	1.71 ± 0.32	1.36 ± 0.38	0.88
	App velocity	−0.14 ± 0.02	0.13 ± 0.04	0.65
	HRC	5.03 ± 0.55	3.86 ± 0.51	0.0001
	MDA	1.23 ± 0.15	1.36 ± 1.47	0.59

ORA	CH	11.44 ± 13.75	8.00 ± 0.86	0.36
	CRF	6.08 ± 1.56	6.4 ± 1.7	0.58

Pentacam	CCT	443 ± 48.26	433 ± 65.58	0.75
	Ks	54.85 ± 3.64	83.52 ± 110.87	0.01
	Kf	50.77 ± 4.26	78.23 ± 117	0.0001

IOP	ORA	12.53 ± 2.22	12.85 ± 3.11	0.77
	Corvis	13.06 ± 1.52	13.75 ± 2.90	0.55

**Table 3:** The results of parameters of the group treated with KeraRing before and after ICRS implantation.

KeraRing		Pre-op	Post-op	*p* value
Number		16	16	

Age		33.86 ± 8.5	33.86 ± 8.5	0.041

CR	Sphere	−0.9 ± 3.23	−0.39 ± 2.87	0.039
	Cylinder	−2.17 ± 5.40	−2.43 ± 4.78	0.59
	Axis	80.57 ± 45.10	89.55 ± 58.43	0.24

Corvis-ST	App time 1	6.96 ± 0.27	7.04 ± 0.61	0.12
	App time 2	21.58 ± 0.43	21.44 ± 0.68	0.13
	App length	1.69 ± 0.30	1.83 ± 0.35	0.51
	App velocity	0.63 ± 1.73	0.11 ± 0.05	0.32
	HRC	6.32 ± 1.53	5.50 ± 1.21	0.01
	MDA	1.09 ± 0.14	1.01 ± 0.23	0.12

ORA	CH	8.78 ± 1.54	7.6 ± 0.73	0.67
	CRF	7.74 ± 2.49	6.64 ± 2.41	0.10

Pentacam	CCT	468.85 ± 41.18	460.91 ± 54.27	0.75
	Ks	49.40 ± 2.93	43.81 ± 2.70	0.34
	Kf	44.75 ± 2.31	81.30 ± 120.87	0.12

IOP	ORA	13.29 ± 3.23	10.43 ± 1.63	0.03
	Corvis	13.70 ± 1.45	12.19 ± 2.66	0.02

**Table 4:** Comparison of the results of KeraRing and MyoRing implantation in KCN patients.

Groups		Post-op MyoRing	Post-op KeraRing	*p* value
Number		16	16	

FCR	Sphere	−0.28 ± 5.26	−0.39 ± 2.87	0.68
	Cylinder	−0.12 ± 2.75	−2.43 ± 4.78	0.12
	Axis	92.59 ± 62.10	89.55 ± 58.43	0.89

Corvis-ST	App time 1	6.98 ± 0.49	7.04 ± 0.61	
	App time 2	21.52 ± 0.72	21.44 ± 0.68	0.76
	App length	1.36 ± 0.38	1.83 ± 0.35	0.15
	App velocity	0.13 ± 0.04	0.11 ± 0.05	0.15
	MDA	1.36 ± 1.47	1.01 ± 0.23	0.20
	HRC	3.86 ± 0.51	5.50 ± 1.21	0.36

ORA	CH	8.00 ± 0.86	7.6 ± 0.73	0.13
	CRF	6.4 ± 1.7	6.64 ± 2.41	0.24

Pentacam	CCT	433 ± 65.58	460.91 ± 54.27	0.26
	Ks	83.52 ± 110.87	43.81 ± 2.70	0.30
	Kf	78.23 ± 117	81.30 ± 120.87	0.11

IOP	ORA	12.85 ± 3.11	10.43 ± 1.63	0.04
	Corvis	13.75 ± 2.90	12.19 ± 2.66	0.02

## Data Availability

The data used to support the findings of this study are available from the corresponding author upon request.
